# Temporal Artery Biopsies: Understanding the Low Positivity Rate

**DOI:** 10.7759/cureus.81714

**Published:** 2025-04-04

**Authors:** Stella-Marie Girard, Caroline François

**Affiliations:** 1 Plastic and Reconstructive Surgery, Christian Cabrol Hospital, Reims, FRA; 2 Plastic and Reconstructive Surgery, Reims Champagne-Ardenne University, Reims, FRA; 3 UR 3081, Reims Champagne-Ardenne University, Reims, FRA

**Keywords:** general plastic surgery, good clinical practices, large-vessel vasculitis and gca, temporal arteritis, temporal artery biopsy

## Abstract

Objective

We compared the yield of temporal artery biopsies with the clinical diagnoses made by referring physicians and determined the factors associated with a positive biopsy.

Methods

This is a monocentric, retrospective analytical study of patients treated between January 2021 and December 2024 who underwent temporal artery biopsy for suspected giant cell arteritis. The primary endpoint was the biopsy positivity rate. We also studied patient-related factors, symptoms, and clinical variables, including surgical and pathological factors, to identify those associated with a positive biopsy result.

Results

The study included 72 patients (40 females, 32 males). General health deterioration (OR = 3.71, *p* = 0.05) was significantly associated with a positive biopsy in the univariate analysis. The average length of the biopsy specimen after fixation was 13.4 mm. The positivity rate of temporal artery biopsies was 16.6% (n=12), while in 55.5% of cases (n=40), referring physicians ultimately diagnosed typical isolated Horton’s disease (n=29, 40.2%) or isolated Horton’s disease associated (n=10, 13.8%) with pseudo-polyarthritis rheumatica.

Conclusion

The anatomic-clinical discordance highlighted in our study supports findings from the literature. This can be explained by factors related to the pathology itself but also by a proactive diagnostic approach and variability in performing the surgical procedure. We have proposed avenues to refine the indications for this procedure and improve the performance of our technique. Temporal artery biopsy should be reserved for well-supported suspicions of Horton’s disease. Sampling upstream from the bifurcation of the superficial temporal artery appears to offer the best reproducibility.

## Introduction

Giant cell arteritis (GCA) is the most common vasculitis affecting the -medium and large-caliber vessels in adults over 50 years of age [1]. Symptoms generally include headaches, decreased visual acuity, jaw claudication, scalp tenderness, inflammatory pain in the girdles, or non-specific symptoms such as fever, weight loss, and asthenia [2]. GCA has a particular tropism for the arteries of the head. Thus, the superficial temporal artery, the terminal branch of the external carotid artery, can be sampled through a direct approach either during its vertical path at its exit from the parotid gland or at its frontal portion after a bifurcation of varying height [3]. This anatomical specificity makes temporal artery biopsy an easily accessible tool for diagnosing GCA. This reference examination for the diagnosis of GCA allows for a definitive diagnosis [4,5]. Histological analysis reveals a segmental and focal involvement of the adventitia, media, and intima (panarteritis) with rupture of the internal elastic lamina and thickening of the intima. The presence of giant cells is observed in only 40% to 50% of cases [5]. Its imperfect sensitivity ranges from 70% to 90%. However, a negative result in this examination should not rule out this pathology, as the diagnosis relies on a combination of clinical, biological, and paraclinical findings. As surgeons, we are frequently asked to perform this procedure, but the histopathological result is often disappointing. The aim of this study is to compare the performance of temporal artery biopsies against definitive diagnoses concluded by our colleagues in internal medicine and rheumatology. We investigate factors that may be associated with a positive biopsy and develop reflections to clarify the indications for this procedure and enhance the performance of our technique.

## Materials and methods

This is a retrospective, analytical, and monocentric study. We included patients for whom we were consulted between January 2021 and December 2024 for a temporal artery biopsy due to suspected GCA. These patients were referred by the departments of internal medicine, infectious diseases, rheumatology, neurology, and geriatrics.

Data collection

Medical records of the patients were reviewed. We collected data on the patients' age, sex, and symptoms (general signs, cephalic symptoms, ophthalmological signs, and rheumatological symptoms). The presence of an inflammatory syndrome was considered based on a C-reactive protein (CRP) level greater than 20 mg/L. The results of various imaging examinations (Doppler ultrasound of the superficial temporal arteries, positron emission tomography [PET], and thoracic CT angiography) were recorded.

Special attention was given to the date of surgery, the side of the biopsy, the level of training of the operators, and the presence and duration of preoperative systemic corticosteroid therapy. The size of the biopsy after fixation, the staining methods used, the findings of the pathologists, and their level of training (junior/senior) were also noted.

**Figure 1:**
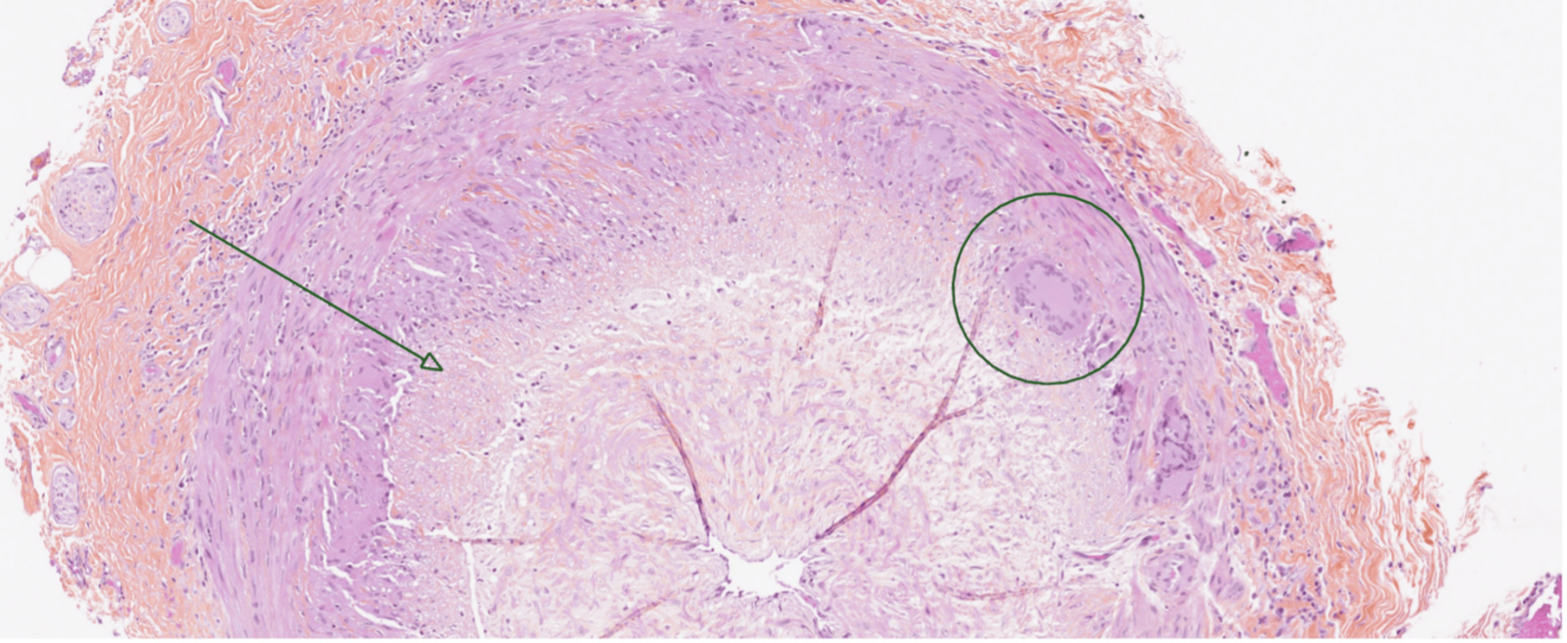
Anatomopathological section of a positive temporal artery biopsy after staining with hematoxylin and eosin, magnified 10x. The arrow indicates a pan-wall inflammatory infiltrate marked by the presence of small lymphocytes (dark purple nuclei) extending from the intima to the adventitia. The circle indicates a multinucleated giant cell. This image has never been used for publication and has been authorized for publication by the Department of Pathology at Centre Hospitalier Universitaire of Reims.

A biopsy was considered positive if the analysis revealed arteritis, defined by the infiltration of mononuclear cells into the arterial wall with interruption of the internal elastic lamina (Figure 1). Normal biopsies or those showing abnormalities not suggestive of arteritis were classified as negative.

The final clinical diagnosis made by the referring physician was determined by reviewing the follow-up reports completed after the initial hospitalization.

Surgical technique

**Figure 2:**
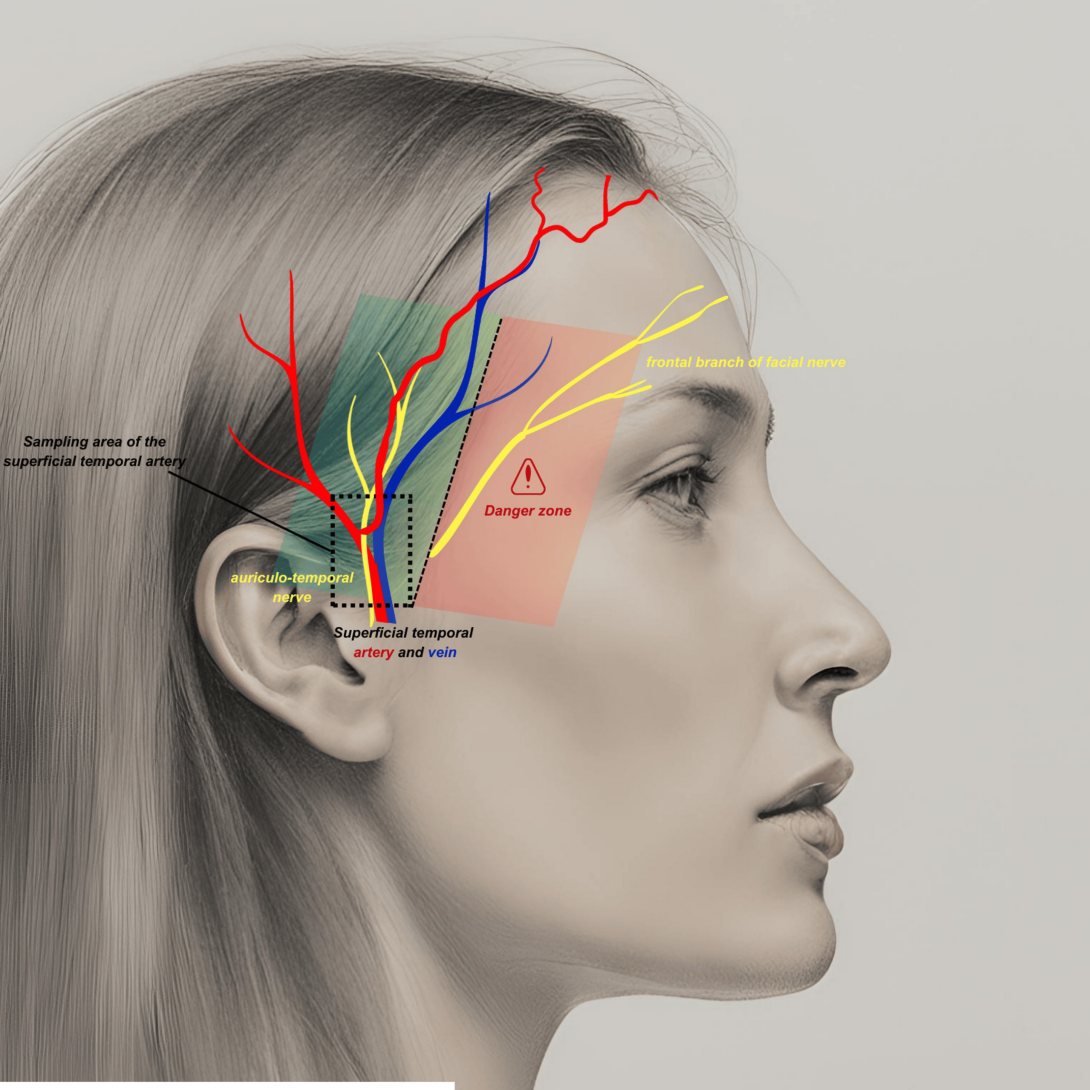
Illustration of the areas of caution during a superficial temporal artery biopsy. The sample is taken above the tragus, in line with the vertical branch of the superficial temporal artery. Caution is exercised with the auriculotemporal nerve, which runs dorsally to the superficial temporal artery, and more anteriorly with the frontal branch of the facial nerve. This image was generated using artificial intelligence using the Canva software, which utilizes advanced algorithms to create visuals based on user inputs and design preferences. The annotations have been added by the author using the same software.

The surgical procedure was performed under local anesthesia. After identifying the path of the superficial temporal artery by palpating the pulse, two different techniques could be used: a temporal-frontal incision over the frontal branch downstream from the zygomatic bifurcation or a pre-tragial approach (Figure 2).

Anatomopathological analysis technique

After being received in the anatomical pathology laboratory and fixed in formalin, the specimen was entirely embedded and examined at multiple levels using hematoxylin and eosin staining. Orcein staining can be used on a case-by-case basis as a complement to highlight thinning or fragmentation of the elastic fibers of the arterial wall.

Statistical analysis

A univariate analysis was conducted to identify factors associated with a positive biopsy. Categorical variables were compared using univariate logistic regressions, and results were expressed in terms of odds ratios (OR). P-values were calculated to assess statistical significance, with a threshold of p < 0.05 considered significant. All statistical analyses were performed using R software Version 2023 (R Foundation for Statistical Computing, Vienna, Austria). The study was registered under the General Data Protection Regulation (GDPR) with the registration number MR004210220252.

## Results

The main results were classified into two groups: positive biopsy and negative biopsy (Table 1).

**Table 1:** Comparison of clinical and paraclinical characteristics between the positive biopsy and negative biopsy groups.

Variables	Biopsy result				Total (n = 72)
	Positive biopsy (n = 12), 16.67%		Negative biopsy (n = 60), 83.33%		
	Frequency	%	Frequency	%	Frequency
Female sex	8	66.7	32	53.3	40 (55.5%)
Male sex	4	33.3	28	46.6	32 (44.4%)
Mean age, years (min-max)	72 (56-84)		73.4 (50-93)		73 (50-93)
Final diagnosis					
Giant cell arteritis (Horton's disease)	9	75	20	33.3	29 (40.2%)
Giant cell arteritis + PMR	2	16.7	8	15	10 (13.8%)
PMR	1	8.3	9	15	10 (13.8%)
Clinical characteristics					
Alteration of general condition	8	66.7	21	35	29 (40.2%)
Unexplained fever	1	8.3	9	15	10 (13.8%)
Headaches	6	50	32	53.3	38 (52.7%)
Scalp hyperesthesia	3	25	16	26.7	19 (26.4%)
Jaw claudication	3	25	16	26.7	19 (26.4%)
Transient visual disturbances	1	8.3	16	26.7	17 (23.6%)
Severe visual disturbances	4	33.3	12	20	16 (22.2%)
Joint symptoms	4	33.3	29	48.3	33 (45.8%)
Temporal pulse abnormalities	5	41.6	11	23.9	16 (22.2%)
Biology					
CRP > 20 mg/L	9	75	48	80	57 (79.1%)
Radiology					
Ultrasound Doppler	11/12		46/60		57/72
Halo sign	6	54.5	9	19.5	15
PET scan	10/12		48/60		58/72
Arterial hypermetabolism	6	60	17	35.4	23
Aortic angioscan	4/12		28/60		32/72
Aortitis	1	25	5	17.8	6
Presence of preoperative corticosteroid therapy	8	66.6	39	65	47
Mean duration of preoperative corticosteroid therapy (days)	10 (min 1; max 34)		14.2 (min 2; max 121)		

A total of 72 patients (40 women, 32 men) were treated for a temporal artery biopsy during this period. The average age of the population was 73 years (minimum 50 years - maximum 93 years) (Table 1). All (100%) of the biopsies were unilateral, while 12 (16.6%) led to a definitive histological diagnosis of GCA. In 54% of patients (n=39), the diagnosis of isolated GCA (n=20, 40%) or GCA associated with polymyalgia rheumatica (n=10, 13.8%) was made during follow-up consultations. The diagnosis of polymyalgia rheumatica without arteritis was made in 10 patients (13.8%).

Clinical signs

The most frequently observed clinical sign in patients with a positive biopsy was a deterioration in general condition (n=8, 66.7%). Headaches were found in one out of two patients (n=6, 50%). Four patients with a positive biopsy (33.3%) presented with severe ophthalmological involvement, such as NOIAA (non-arteritic anterior ischemic optic neuropathy) or OACR (optic artery central retinal) occlusion. We observed jaw claudication and scalp hyperesthesia in three patients each in the positive biopsy group (25%). Among the patients whose biopsy was negative, the most frequently observed clinical sign was headaches (n=32, 53.3%), followed by joint symptoms (n=29, 48.3%) and deterioration of general condition (n=21, 35%).

Corticosteroid therapy

In the positive biopsy group, preoperative corticosteroid therapy was administered in eight (66.6%) patients. The average delay before starting the treatment in this group was 10 days (minimum 1; maximum 34). In the negative biopsy group, corticosteroid therapy was started preoperatively in 39 (65%) patients. The average delay in this group was 14.2 days (minimum 2 days; maximum 121 days).

Surgical procedure

Eleven operators participated in performing these biopsies: three residents in advanced training, four junior doctors, and four senior doctors. One patient in 10 (n=55) was operated on by a resident, and more than three-quarters (n=55) were operated on by a junior doctor.

Complications

One postoperative complication was observed three weeks postoperatively in the form of dysesthesia in the area of the temporal artery biopsy.

Anatomopathological analysis

The histological analysis was performed by 16 anatomic-pathologists. Among them, 6 were junior doctors and 10 were senior doctors. The average length of the specimen after fixation was 13.4 millimeters. The data from the anatomic-pathological analysis are shown in Table 2.

**Table 2:** Comparison of histological characteristics between the positive biopsy and negative biopsy groups.

Variables	Biopsy result			
	Positive biopsy (n =12), 16.67%		Negative biopsy (n = 60), 83.33%	
	Frequency	%	Frequency	%
Wall inflammation	12	100	0	0
Internal elastic lamina fragmentation	10	83	3	0.05
Giant cells	10	83	0	0
Medial calcification	0	0	6	0.1
Arteriosclerosis	0	0	4	0.06
Fibrosis	0	0	3	0.05
Avascular sample	0	0	3	0.05
Orcein staining	6	50	22	36.6
Junior doctor	5	41.6	14	23.3
Senior doctor	7	58.3	46	76.6
	Length (mm)		Length (mm)	
Size after fixation	13.5 (min 8; max 18)		13.4 (min 6; max 25)	

Differential diagnoses

Among the patients with a negative biopsy, more than one-third (n= 21, 35%) were ultimately diagnosed with different pathologies based on clinical evolution or results from other paraclinical tests. Atheromatous disease (two cases), rheumatoid arthritis (two cases), Arnold's neuralgia (one case), meningeal hemorrhage (one case), idiopathic chronic intermittent fever (one case), and Behçet's syndrome (one case) were found. No diagnosis could be established in 13 (18%) patients.

Factors predisposing to a positive biopsy

Univariate analysis of factors associated with a positive biopsy is summarized in Table 3. Univariate analysis identified several factors associated with a positive biopsy. Altered general condition (AEG) was significantly associated with an increased chance of a positive biopsy (OR = 3.71, p = 0.05), as was the presence of a halo sign on Doppler ultrasound (OR = 5.66, p = 0.01). Although severe vision loss (NOIAA/OACR) showed a trend toward association with a positive biopsy (OR = 2.00), this association was not statistically significant (p = 0.32). Other variables studied, such as sex (OR = 0.57, p = 0.4), fever (OR = 0.52, p = 0.55), headaches (OR = 0.88, p = 0.83), hypermetabolism (OR = 2.53, p = 0.15), and a good response to corticosteroids (OR = 4.00, p = 0.35), did not show a significant association with a positive biopsy.

**Table 3:** Univariate analysis of factors associated with a positive biopsy.

Variable	OR	p-Value
Sex (male)	0.57	0.4
Anatomopathology status (senior)	0.43	0.2
General condition deterioration	3.71	0.05
Fever	0.52	0.55
Headache	0.88	0.83
Scalp hyperesthesia	0.92	0.9
Jaw claudication	0.92	0.9
Transient visual disturbances	0.25	0.2
Sever visual disturbances	2.0	0.32
Presence of arthralgia	0.53	0.35
Biological inflammatory syndrome (CRP ≥ 20 mg/L)	0.75	0.7
Halo sign on temporal artery ultrasound	5.66	0.01
Response to corticosteroids (good)	4.0	0.35
Arterial hypermetabolism on PET-CT	2.53	0.15

Factors predisposing to a negative biopsy

Univariate analysis did not show a significant association between the duration of corticosteroid therapy and negative temporal artery biopsy results (OR = 0.96; 95% CI: 0.85-1.02; p = 0.45). Similarly, the size of the specimen after fixation was not significantly associated with negative biopsies (OR = 1.01; 95% CI: 0.87-1.16; p = 0.92).

## Discussion

The median yield of temporal artery biopsy in the literature is 25%, and there is significant heterogeneity between centers [6,7]. In our study, only 12 of the biopsies performed (16.6%) were positive. The diagnosis of GCA in the hospital involves four specialists: physician (internist or rheumatologist), radiologist, surgeon, and pathologist. Cohesion and communication are fundamental. We propose some avenues for reflection to understand these numbers and improve the yield of this procedure, which is the result of multidisciplinary work.

Patient selection

Demographics and Indicative Signs

GCA primarily affects people over the age of 60 years, with a peak prevalence at around 70 years. It is more frequent in women, in whom the incidence is two to three times higher [8]. In our study, 55% of the requests were for women (n = 40) and 66% of the positive biopsies were found in women (n=8).

The symptoms of GCA can be nonspecific outside of a typical presentation. Headaches were only observed in one in two patients whose biopsy was positive. The most frequently observed clinical sign in patients with a positive biopsy was a general deterioration in health, which was also significantly associated with biopsy positivity.

Too Easy Access?

Access to temporal artery biopsy in a teaching hospital is relatively easy. The procedure is quick, taking between 20 and 40 minutes, and low-cost [9]. Postoperative complications are rare and generally benign (bleeding, local infection, sensory disturbances). These characteristics may explain the sometimes very proactive attitude of prescribing physicians. In some cases, the goal is not so much to confirm GCA as to rule it out. Faced with ambiguous symptoms and inconclusive paraclinical tests, it is difficult to definitively reject the diagnosis of GCA. On the other hand, a missed diagnosis can have dramatic consequences such as irreversible blindness [6]. Therefore, there is a favorable benefit-risk ratio that sometimes justifies performing this seemingly "futile" procedure. It remains essential to inform internists about the invasive nature of this intervention and its psychological impact on the patient.

Other Useful Tests for Diagnosis

The use of radiological examinations has been unequal: one in five patients (n=15, 20.8%) did not benefit from a Doppler ultrasound of the temporal arteries or a PET scan (n = 14, 19.4%), and more than half of the patients did not undergo an angioscan of the aorta and supra-aortic trunks (n= 40, 55.5%).

There are no precise recommendations regarding the use of PET scan or CT angiography, with their indication remaining at the discretion of the clinician [9]. However, according to the recommendations of EULAR (European Alliance of Associations for Rheumatology), color Doppler ultrasound of the temporal arteries and supra-aortic trunks should be performed as the first-line investigation in suspected GCA due to its low invasiveness and reduced cost. Moreover, it has a lower false-negative rate than temporal artery biopsy [10]. In our series, among the patients with a positive biopsy, nearly half did not present the halo sign (n=6, 45%). This may be explained by corticosteroid therapy, which can make this sign disappear after five days [9].

The sensitivity and specificity of Doppler ultrasound of the temporal arteries can be limited due to its operator-dependence. Therefore, it is crucial that the examination be performed by an experienced operator with appropriate equipment. In the absence of these resources, referring physicians may easily turn to temporal artery biopsy. Like temporal artery biopsy, Doppler ultrasound of the temporal arteries is particularly effective for diagnosing cranial GCA. Therefore, in line with EULAR recommendations, this examination may suffice to confirm the diagnosis of GCA when the clinical probability is high. On the other hand, it can also be used to exclude the diagnosis of GCA when the probability is low, for instance, in cases of nonspecific clinical signs and in the absence of an inflammatory syndrome.

Temporal artery biopsy

Preoperative Corticosteroid Therapy

The average duration of preoperative corticosteroid therapy was 15 days in the negative group versus 10 days in the positive group, without a statistically significant association. This delay may be linked to issues with access to operating rooms. It is also plausible that in some cases, temporal artery biopsy was only considered after an initial phase of clinical uncertainty.

It is now established that histological abnormalities of the temporal artery can persist for at least 15 days after the initiation of systemic corticosteroid therapy [9]. This delay may be extended up to four weeks even with high-dose corticosteroid therapy [11,12]. In the context of arteritis secondary to conditions such as polymyalgia rheumatica (PMR), which is treated with low-dose corticosteroids, a late temporal artery biopsy can still provide useful diagnostic information [12]. However, in the case of suspected GCA and in the absence of severe signs, temporal artery biopsy is preferred before initiating corticosteroid treatment [9].

Surgical Technique

The temporal artery biopsy is a surgical procedure that appears simple and is often performed by young operators and interns. More than three-quarters of the procedures (n=55) are performed by junior doctors. This crucial procedure in the management of GCA must be performed with care, as it is not without risks, particularly damage to the frontal branch of the facial nerve [12]. Therefore, training in this technical procedure is essential and should be done through hands-on training in the operating room and through educational videos.

Two incision techniques, pre-tragal and frontal, were used, but the type of incision was not systematically specified in the operative reports, which prevented an evaluation of the impact of the biopsy site on the biopsy yield. Although postoperative complications are rare, this low incidence of complications could be attributed to the absence of systematic postoperative consultations. It is useful to train physicians who follow up with these patients to look for complications using a checklist.

The technique described by Cahais et al. appears to be the most reproducible technique [3]. Anatomical variations exist, particularly at the bifurcation of the temporal artery into the frontal and parietal branches [13]. This method allows for the harvesting of a segment of the temporal artery in its constant portion. It is relatively simple, as the superficial temporal artery is easily identifiable through an incision at the pre-tragal fold. A 3-cm incision is made, ensuring not to descend below the tragus to avoid damaging the intra-parotid portion of the artery. It is crucial not to confuse the artery with the superficial temporal vein (which runs ventral to the artery) and the auriculotemporal nerve, which runs dorsal. The dissection of the artery should be atraumatic.

The area of risk corresponding to the frontal branch of the facial nerve can be identified by drawing an axis: an invisible line between the outer canthus and the tragus, which helps visualize the course of the nerve and ensures the safety of the procedure. (Figure 2) [14]. A pulse at the level of the artery, even a minimal one, should be visualized if the anatomical structures are not clear. An avascular sample is regrettable and should prompt discussion of a contralateral sample [15].

The length of the artery segment to be harvested is a subject of debate, and there is no consensus. We did not find any association between the length of the harvested segment and the negativity of the biopsy. Larger studies show no significant difference in positivity rates between samples of less than 1 cm and those longer than 2 cm [16-18]. Some authors suggest that a minimum length of 0.5 cm for the harvested segment, after fixation, is sufficient, taking into account an average loss of 2.4 mm after the sample is fixed [18].

Anatomopathological Analysis

In our series, 16.6% of the samples (n=12) showed atypical findings such as fibrosis, medial calcinosis, or isolated fragmentation of the internal elastic lamina. According to Muratore et al., the nonspecific abnormalities found by histologists, such as intimal hyperplasia, calcifications, and isolated fragmentation of the elastic lamina, do not allow differentiation between patients with GCA and those without GCA [19]. These authors recommend avoiding the term "healed arteritis" and reserving the diagnosis of arteritis for biopsies that show inflammation. In our study, a varied number of pathologists (16 in total) analyzed the samples, with heterogeneity in their level of training, which could influence the interpretation of the results. Similarly, orcein staining was only used in 37.8% of the cases (n=28). This raises concerns about the need for a standardized protocol in anatomical pathology for the analysis of these samples.

Final diagnosis

Our study reveals an anatomical diagnostic discordance, as despite a low positivity rate, GCA was diagnosed in 54% of patients (n=39), with 38.9% of them (n=28) in the negative biopsy group. Although this final diagnosis was made by an experienced internist or rheumatologist with sufficient clinical experience, histologically unconfirmed Horton’s disease remained a presumptive diagnosis.

Diagnosis of Giant Cell Arteritis in 2022

The American College of Rheumatology’s (ACR) 2022 guidelines reiterate the diagnostic criteria for GCA [20]. They specify that a "positive temporal artery biopsy or a halo sign on ultrasound" contributes to the diagnostic algorithm with an added value of 5 points. In cases where ultrasound has already revealed a halo sign, performing a temporal artery biopsy is not necessary. The diagnosis of GCA can be confirmed if the total score reaches or exceeds 6 points.

Some individual studies have shown a decrease in the proportion of positive temporal artery biopsies over time. This trend, also observed in our study, could indicate increased confidence among clinicians in diagnosing GCA based on clinical criteria, as allowed by the ACR diagnostic algorithm, even in the absence of a positive biopsy [21].

Differential Diagnoses

In eight (11.1%) patients, the diagnosis of GCA was revised in favor of another diagnosis, with distinct treatment and outcomes. On the other hand, for the 13 (18%) patients for whom no diagnosis could be established after the acute episode, this uncertainty represents a difficult trial for the patients and a source of frustration for the clinician.

Take-home messages

The absence of an inflammatory syndrome makes the diagnosis of GCA unlikely; in such cases, it is preferable to perform a Doppler ultrasound rather than a temporal artery biopsy (TAB) to rule out the diagnosis.

Doppler ultrasound should be performed by an experienced operator as soon as GCA is clinically suspected, either before or just after the initiation of corticosteroid treatment, prior to considering a TAB.

A TAB is unnecessary when a positive halo sign is present in a clinically suggestive context.

In cases of suspected GCA and the absence of severe symptoms, a TAB should be performed before initiating corticosteroid therapy.

Standardization of the surgical technique is essential. We recommend harvesting the artery from its vertical portion, with particular attention to the auriculotemporal nerve and the frontal branch of the facial nerve.

We suggest a minimum biopsy length of 1 cm to compensate for tissue shrinkage due to fixation.

Only the presence of a mononuclear inflammatory infiltrate in the media and/or intima confirms the diagnosis of GCA.

The diagnosis of GCA should be based on the updated EULAR score. GCA is confirmed when the score reaches or exceeds 6 points.

## Conclusions

The positivity rate of the temporal artery biopsy in our series was 16.6% (n=12). Factors associated with a positive biopsy included the deterioration of the patient's general condition and the presence of a halo sign on Doppler ultrasound of the temporal arteries. Although the temporal artery biopsy is a readily accessible procedure, it should be reserved for cases where the suspicion of GCA is well-founded. Its use as a diagnostic exclusion tool can cause psychological distress for the patient, with a hypothetical diagnostic benefit.

It is essential to standardize clinical practices and the management algorithm, from patient admission to the anatomical pathology laboratory, including the operating room. In a surgical department, the uniformity of the procedure is crucial to improve the yield of the intervention and reduce inter-operator variability.
